# A new treatment: hybrid endoscopic ultrasound guided vascular intervention for huge duodenal varices

**DOI:** 10.1055/a-2747-4769

**Published:** 2025-12-11

**Authors:** Kazunori Nagashima, Ryota Matsukawa, Yuya Kumano, Yugo Tetsuka, Tsunehiro Suzuki, Manabu Misu, Atsushi Irisawa

**Affiliations:** 1 12756 Department of Gastroenterology, Dokkyo Medical University School of Medicine, Shimotsuga, Japan; 2 46624 Japanese Red Cross Ashikaga Hospital, Ashikaga, Japan

In recent years, endoscopic ultrasound-guided vascular intervention (EUS-VI) has been developed 1 2 3 . However, conventional EUS-VI has some problems 4 5 . It can only deploy coils at the puncture site. For this reason, it cannot perform selective and multiple embolization for vessels. Furthermore, it presents risks of coil kinking in the needle. This report describes a new treatment: hybrid EUS-VI. This method, which uses a guidewire and microcatheter, can facilitate without the difficulties described above. To the best of our knowledge, this is the first video case of hybrid EUS-VI.

We present a video of a typical case ( Video 1 ). A patient, a 65-year-old man, had alcoholic cirrhosis and huge duodenal varices ( Fig. 1 ). Contrast-enhanced computed tomography showed duodenal varix hemodynamics ( Fig. 2 ). The duodenal varices were punctured using a 19G FNA needle (EZ shot3 plus; Olympus Corp.) at the blood drainage route side. After a 0.018-inch guidewire (Radifocus Guide Mire M; Terumo Corp.) was inserted, the needle was removed. Then, a 2.0-French microcatheter (Headway Plus; Terumo Corp.) was placed along the guidewire ( Fig. 3 ). The blood drainage route was embolized in combination with coils (AZUR; Terumo Corp.) and injection of 25% cyanoacrylate (CA). Similarly, the duodenal varices were punctured at the feeder side. A sclerosant (ethanolamine oleate, EO) was injected into the feeder of the duodenal varices ( Fig. 4 ). Subsequently, we inserted a 0.035-inch coil and 25% CA into the feeder ( Fig. 5 ). Eventually, the blood flow ceased.

This report is a new treatment for EUS-guided vascular intervention using a guidewire and microcatheter in combination. This hybrid EUS-VI is useful and applicable for vascular procedures of EUS-VI.Video 1

**Fig. 1:**
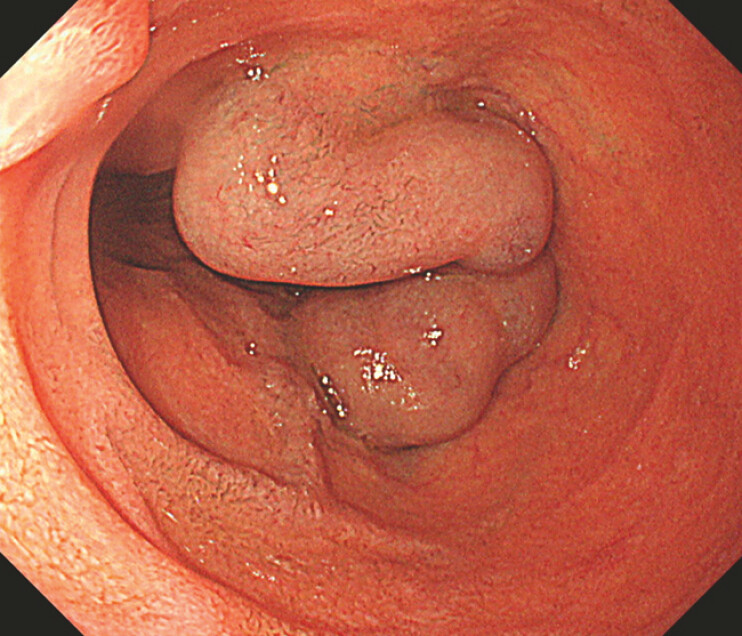
Duodenal varices were thick, showing strong development.

**Fig. 2:**
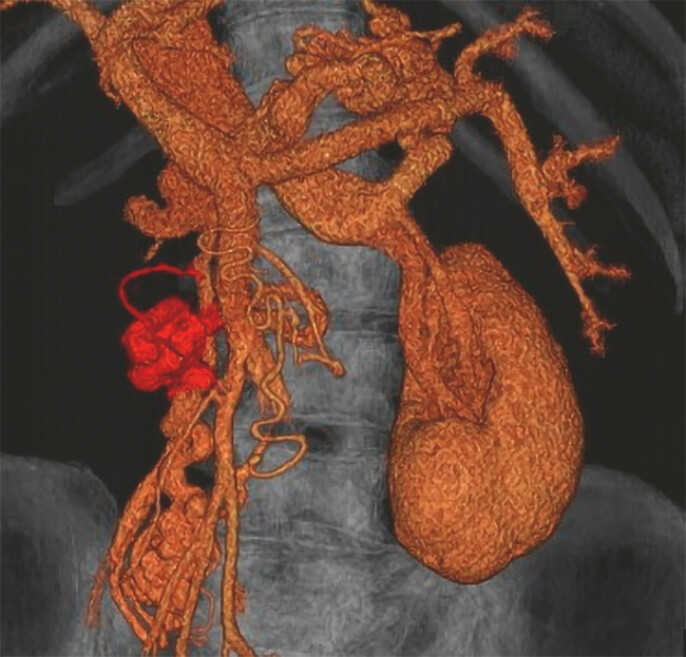
3D-CT showed duodenal varix hemodynamics that fed from the mesenteric vein to the right testicular vein.

**Fig. 3:**
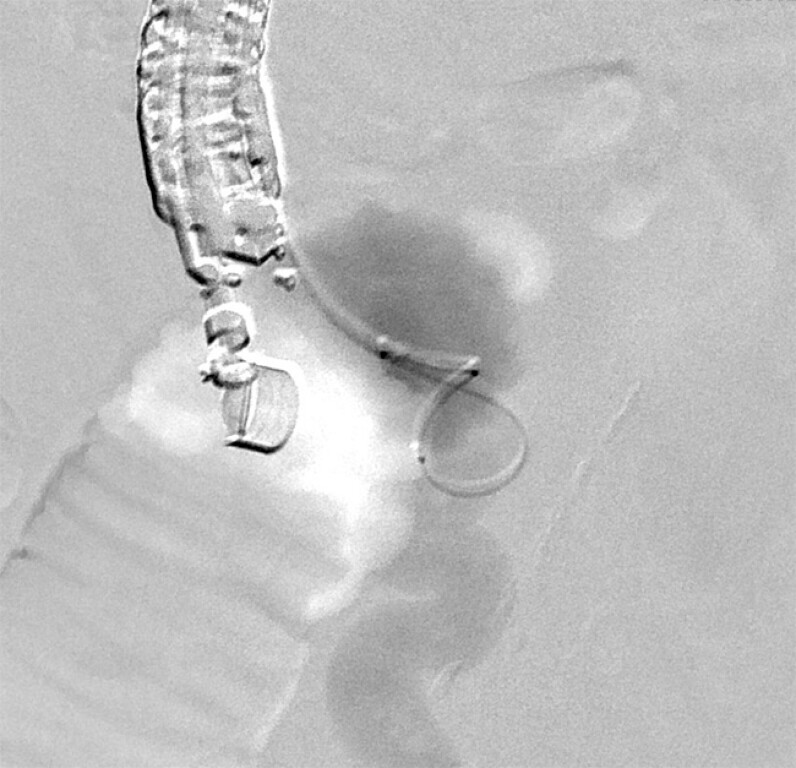
The microcatheter was placed successfully from the varices toward the drainage pathway.

**Fig. 4:**
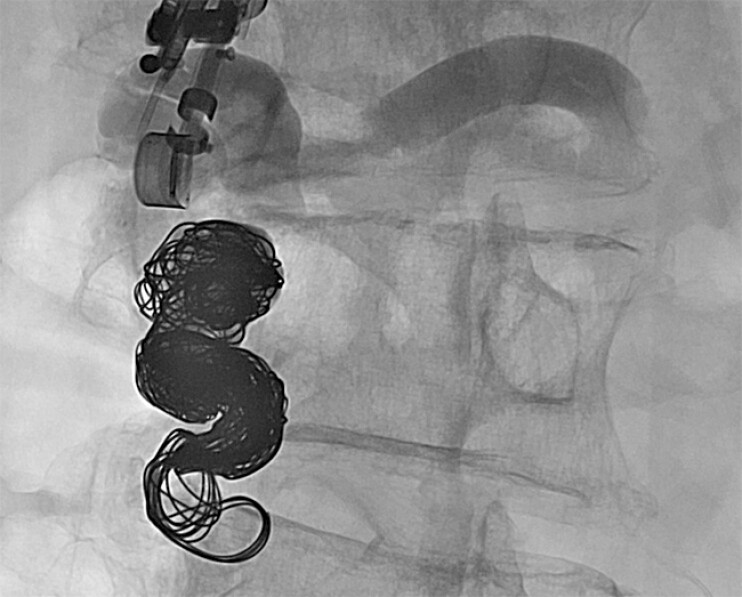
After the drainage pathway was embolized, a sclerosant (ethanolamine oleate, EO) was injected for the feeder of duodenal varices.

**Fig. 5:**
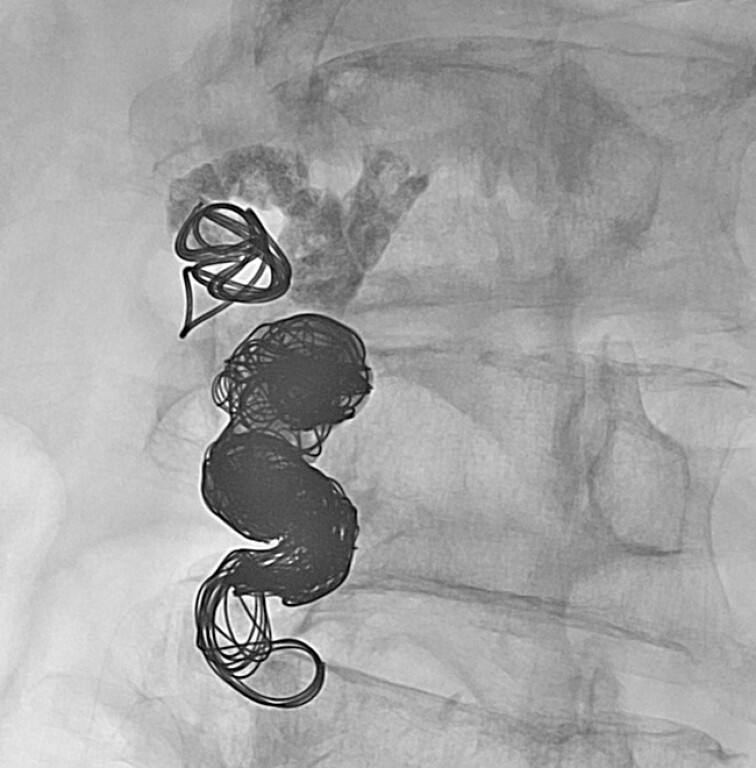
After a 0.035-inch coil was placed, 25% of CA was injected into the feeder.

We developed a new treatment: hybrid EUS-VI. This method can perform selective and multiple embolization for blood vessels without coil kinking. Additionally, this method when used with fluoroscopy allows access to more distant vessels that cannot be visualized using EUS alone. We consider hybrid EUS-VI to be useful and adaptable not only for varices but also for various other vascular procedures of EUS-VI.

Endoscopy_UCTN_Code_TTT_1AS_2AG

## Notes

Endoscopy E-Videos https://eref.thieme.de/e-videos
*E-Videos* is an open access online section of the journal *Endoscopy* , reporting on interesting cases and new techniques in gastroenterological endoscopy. All papers include a high-quality video and are published with a Creative Commons CC-BY license. Endoscopy E-Videos qualify for HINARI discounts and waivers and eligibility is automatically checked during the submission process. We grant 100% waivers to articles whose corresponding authors are based in Group A countries and 50% waivers to those who are based in Group B countries as classified by Research4Life (see: https://www.research4life.org/access/eligibility/ ). This section has its own submission website at https://mc.manuscriptcentral.com/e-videos .
